# Use of medroxyprogesterone acetate in women with ovarian endometriosis undergoing controlled ovarian hyperstimulation for in vitro fertilization

**DOI:** 10.1038/s41598-017-12151-7

**Published:** 2017-09-20

**Authors:** Haiyan Guo, Yun Wang, Qiuju Chen, Weiran Chai, Lihua Sun, Ai Ai, Yonglun Fu, Qifeng Lyu, Yanping Kuang

**Affiliations:** Department of Assisted Reproduction, Shanghai Ninth People’s Hospital, Shanghai Jiaotong University School of Medicine, Shanghai, 200011 China

## Abstract

This study investigated the use of medroxyprogesterone acetate (MPA) or a short protocol for controlled ovarian hyperstimulation (COH) in patients with advanced endometriosis who have normal ovarian function, and to compare cycle characteristics and pregnancy outcomes after frozen-thawed embryo transfer (FET). This was a retrospective case-control study of 244 patients with advanced endometriosis undering COH. The patients were allocated to three groups: the surgery group with MPA COH (62 patients, 71 IVF/ICSI cycles, 78 FET cycles); the aspiration group with MPA COH (85 patients had ovarian “chocolate” cysts (>3 cm) aspirated, 90 IVF/ICSI cycles, 76 FET cycles); and the short protocol group (97 patients, 101 IVF/ICSI cycles, 51 FET cycles). The results showed that higher rates of mature oocyte, D3 high quality embryo, hMG dose were observed in the two study groups using MPA compared with the short protocol. The number of >10–14 mm follicles on the trigger day, D3 top-quality embryos, viable embryos, rates of cancellation, fertilization, implantation, pregnancy outcomes were similar among the three groups. The oocytes, embryos, and pregnancy outcomes were not influenced by endometrioma surgery or presence of endometrioma. MPA COH could be effective for women with ovarian advanced endometriosis who had normal ovarian function.

## Introduction

Endometriosis is an estrogen (E_2_)-dependent condition characterized by endometrial tissue located outside of the uterus. It affects approximately 10% of women in the United States and 20–40% of women seeking infertility evaluation^1–3^. Even mild endometriosis may have a direct negative effect on fertility because of its impact upon oocyte development, embryogenesis, or implantation^4–9^. Nevertheless, the exact mechanisms are unknown^5^.

During IVF cycles, controlled ovarian hyperstimulation (COH) using gonadotropin-releasing hormone agonist (GnRH-a) may suppress some of the negative effects of endometriosis on pregnancy^10^. Prolonged GnRH-a treatment prior to IVF may improve fertility rates in advanced endometriosis^11,12^, but supra-physiological concentrations of E_2_ and progesterone could affect endometrium receptivity and pregnancy outcomes of patients with endometriosis^13,14^. Whether women with endometriosis have a reduced pregnancy rate compared with women with tubal factor infertility^15–18^ or not^19–25^ remains controversial.

Progestins have been used for endometriosis therapy for more than 40 years. It is believed that they act as progesterone receptor agonists, but their pharmacological actions are still not understood^9,26^. Progestins create a low E_2_ environment and inhibit the growth of ectopic endometrium. Progestins have good tolerability, minor metabolic effects, and low cost. Fechner *et al*.^27^ suggested that progestins can regulate local E_2_ biosynthesis in women with endometriosis. Progestin may yield a greater proportion of mature oocytes capable of fertilization and cleavage compared with GnRHa and a low dose of hCG^28^, but R5020 used in this previous study^28^ is known to have specific characteristics not shared by other progestins. Restoration of meiosis in oocytes is triggered by steroid hormones, specifically progestin^29^. High ratios of progestin to E_2_ in follicular fluid are associated with better embryo development^30^.

Medroxyprogesterone acetate (MPA) is considered as an alternative treatment to progestins because MPA has moderate to strong progestin action, less androgenic properties, and does not interfere with measurement of endogenous progestin production^31^. Previous studies described the use of MPA in patients undergoing COH for IVF^32,33^.

Progesterone can prevent moderate/severe ovarian hyperstimulation syndrome (OHSS) during COH in normal women and patients with polycystic ovarian syndrome (PCOS)^32–34^. MPA is an effective oral alternative for the prevention of a premature luteinizing hormone (LH) surge in woman undergoing COH, and the pregnancy outcomes of frozen-thawed embryo transfer (FET) indicated that the embryos originating from MPA co-treatment with human menopausal gonadotrophin (hMG) had similar developmental potential as the short protocol^32^.

The aim of this study was to explore the possibility of using MPA with hMG in advanced endometriosis during COH in IVF. The study investigated the cycle characteristics and endocrine profiles resulting from using MPA cotreatment in patients with advanced endometriosis taking gonadotropin and who were undergoing IVF/ICSI treatments. The pregnancy outcomes were compared with FET cycles in patients with ovarian endometriosis and using the short protocol.

## Methods

### Patients

This was a retrospective case-control study of patients with advanced endometriosis induced with the MPA protocol (cases) vs. the short protocol (controls). All cycles were performed at the Department of Assisted Reproduction of Shanghai Ninth People’s Hospital, Shanghai Jiaotong University School of Medicine, from November 2015 to October 2016. The study protocol was approved by the ethics committee of Shanghai Ninth People’s Hospital. All procedures performed in studies involving human participants were in accordance with the ethical standards of the institutional research committee and with the 1964 Helsinki declaration and its later amendments or comparable ethical standards. All participants provided informed consent after counseling for infertility treatments and routine IVF procedures.

The inclusion criteria were: (1) 20–40 years of age; (2) had regular ovulation; (3) follicle stimulating hormone (FSH) levels < 10 IU/L; 4) antral follicle count (AFC) of 5–20; (5) advanced endometriosis as determined by laparoscopy or laparotomy and staged III-IV according to the revised American Fertility Society (AFS) classification of endometriosis^35^; and (6) had ovarian endometriomas resected by laparoscopy or laparotomy before IVF or had ovarian endometriomas cysts that were aspirated under transvaginal ultrasound guidance and identified as “chocolate” cysts (>3 cm) during ovulation monitoring or at the time of oocyte retrieval. The exclusion criteria were: (1) polycystic ovarian syndrome (PCOS); (2) hydrosalpinx; (3) adenomyosis (AM) diagnosed by laparoscopy or laparotomy, disordered myometrial echo confirmed by ultrasound examination, or mild adenomyosis diagnosed by MRI; (4) documented ovarian failure including basal FSH above 10 IU/L; (5) clinically significant systemic disease such as renal failure; (6) had received hormonal treatments in the previous 3 months; or (7) any contraindications to ovarian stimulation treatment.

In the surgery group, 37.1% (23/62) of the patients who underwent laparoscopy or laparotomy had unilateral ovarian endometriomas (>3 cm) before IVF and 62.9% (39/62) had bilateral ovarian endometriomas (>3 cm) before IVF. Twenty patients had cysts recurrence after surgery and 32.3% (20/62) of the patients were treated with peritoneal endometriosis electrocoagulation.

In the aspiration group, 29.4% (25/85) of the patients had unilateral ovarian endometriomas (>3 cm, single or multiple) before IVF, while 71.6% (60/85) had bilateral ovarian endometriomas (>3 cm, single or multiple) before IVF. Before oocyte retrieval, the suspected endometrioma was aspirated with a single puncture. The aspirated material was examined for cytological diagnosis, and no malignancy was found in all cases. If the aspirated fluid of the cyst was chocolate-colored, dense, and contained mucous, the cyst was diagnosed as an endometrioma. Alternatively, when the fluid was pale, serous, and not thick, the cyst was diagnosed as a functional cyst that may have been induced by a previous ovarian hyperstimulation regimen.

### Study design

A total of 262 cycles in 244 patients were analyzed. The cycles were divided into three groups: the surgery group (71 cycles in 62 patients), which included women diagnosed with advanced endometriosis by laparoscopy or laparotomy who had ovarian endometriomas that were all treated surgically before IVF; the aspiration group (90 cycles in 85 patients), which included women who had ovarian endometriomas that were aspirated and identified as “chocolate” cysts (>3 cm) during ovulation monitoring or at the time of oocyte retrieval; and the short protocol group, which included 97 patients with advanced endometriosis using the short protocol (101 IVF/ICSI and 51 FET cycles).

### Ovarian stimulation and embryo culture

The patients in the three groups were given hMG (Anhui Fengyuan Pharmaceutical Co, China) at a dose of 150–225 IU/day and MPA (Beijing ZhongXin Pharmaceutical, China) 4 or 10 mg/day from day menstrual cycle day (MC) 3 of menstruation, after that ultrasound and blood test confirmed the presence of a baseline hormone profile. A short protocol was used for the control group. Patients were administered triptorelin 0.1 mg daily beginning on MC 2 and hMG 150 to 225 IU daily beginning on MC 3. The final stage of oocyte maturation was triggered when there were more than 3 dominant follicles >18 mm in diameter. All follicles with diameters greater than 10 mm were retrieved. Follicular monitoring was started on MC 9 to 11 and was performed every 2 to 3 days using a transvaginal ultrasound to record the number of developing follicles. Serum FSH, LH, E_2_, and P levels were measured using patient blood tests on the same days as the ultrasound exams. The dose of hMG was adjusted according to the estradiol concentrations and ovarian response. In the short protocol group, the final stage of oocyte maturation was triggered using hCG 2000 IU (Lizhu Pharmaceutical Trading Co., Zhuhai, China). A previous study by our group^32^ showed that co-triggering with GnRHa 0.1 mg and a low dose of hCG (1000 IU) had better oocyte maturation than triggering with GnRHa alone in the MPA cotreatment with gonadotropin in a general population of infertile women. Therefore, when three dominant follicles reached 18 mm in diameter, the final stage of oocyte maturation was co-triggered by decapeptyl (0.1 mg) (Ferring International Center SA, Germany) and hCG 1000 IU (Lizhu Pharmaceutical Trading Co, China). In our previous studies, the cycle characteristics were similar when triggered by GnRH-a or HCG^34,36^. Transvaginal ultrasound-guided oocyte retrieval was performed 36 to 37 h after triggering. Oocytes were fertilized using either conventional IVF or intra-cytoplasmic sperm injection (ICSI). Examination of embryo quality included the number/uniformity of blastomeres and the degree of fragmentation^37^. Embryo morphology was scored according to the criteria of Cummins^37^. All the highest quality embryos (including at least 6 blastomeres and fragmentation < 50%) were frozen by vitrification on the third day after oocyte retrieval. The embryos that were not of the highest quality were placed for further extended culture until the blastocyst stage. During this stage, only good morphology blastocysts were cryopreserved. Thawed embryos were considered to have survived if 50% of the blastomeres were intact. Only embryos that survived were transferred.

### Transfer of cryopreserved-thawed embryos

Natural FET cycles were used for women with regular menstrual cycles. Letrozole was used and, if necessary, hMG to stimulate monofollicular growth. Letrozole 5 mg was administered from day 3 to 7, and then follicle growth was monitored beginning on day 10. Sometimes treatment included a low dose of hMG (75 IU/day) to stimulate follicular and endometrial lining growth. Administration of 5000 IU of hCG and the timing of FET were performed as previously described^38^. In the present study, there were no differences among groups in pregnancy outcomes, similar to a previous study^39^. A hormone substituted cycle was performed for patients with thin endometria during natural or stimulation cycles with daily oral administration of ethinylestradiol (EE) (Shanghai Xinyi Pharmaceutical, Shanghai, China) 75 µg/ day from day 3 to attain an endometrial thickness of 8 mm. At that time, patients were given 0.4 g intravaginal progestin (LaboratoiresBesins-Iscovesco, France) and estradiol valerate (Abbott Biological B.V, Netherlands) 8 mg daily. The maximum number of transferred embryos was two per patient. The progesterone supplementation was continued until 10 weeks of gestation after pregnancy was achieved.

### Data collection

The number of oocytes retrieved, number of mature oocytes (MII oocyte) retrieved, duration of gonadotropin stimulation, total dose of hMG, cycle cancellation rate, fertilization rate, implantation rate, clinical pregnancy rate, and ongoing pregnancy rate were recorded. Clinical pregnancy and ongoing pregnancy were considered in the presence of a gestational sac with fetal heart activity, as assessed by ultrasound at 7 and 12 weeks of gestation, respectively. The implantation rate was defined as the number of gestational sacs divided by the number of embryos transferred. The early miscarriage rate was defined as the proportion of patients with spontaneous pregnancy termination before the gestational age of 12 weeks.

### Hormone measurement

Serum FSH, LH, E_2_, and progesterone were measured on day 3 of the stimulation cycle, the trigger day, and the day after trigger (12 h later after the injection of GnRHa and hCG). Hormone levels were measured by chemiluminescence (Abbott Biologicals B.V, Netherlands). The upper limit of E_2_ measurement was 5000 pg/mL. The lower limits of detection were: FSH = 0.06 IU/L, LH = 0.09 IU/L, E2 = 10 pg/mL, and P = 0.1 ng/mL.

### Statistical analysis

Continuous data were tested for normal distribution using the Kolmogorov-Smirnov test;those with normal distribution were presented as mean ± standard deviation (SD), while those with non-normal distribution were expressed as median (range or interquartile range). The data was then analyzed using the Kruskal-Wallis test, and Mann-Whitney U test. The post hoc Bonferroni method was used for the comparison of hormone levels at different points in time. The Mann-Whitney U-test was used for the variables with non-normal distribution. P < 0.05 was considered statistically significant. All data were analyzed using the SPSS 16.0 for Windows (IBM, Armonk, NY, USA). Binary logistic regression analysis was performed with clinical pregnancy as the dependent variable, and all of the other measured variables as the independent variables.

## Results

### Patient characteristics

**Figure 1:**
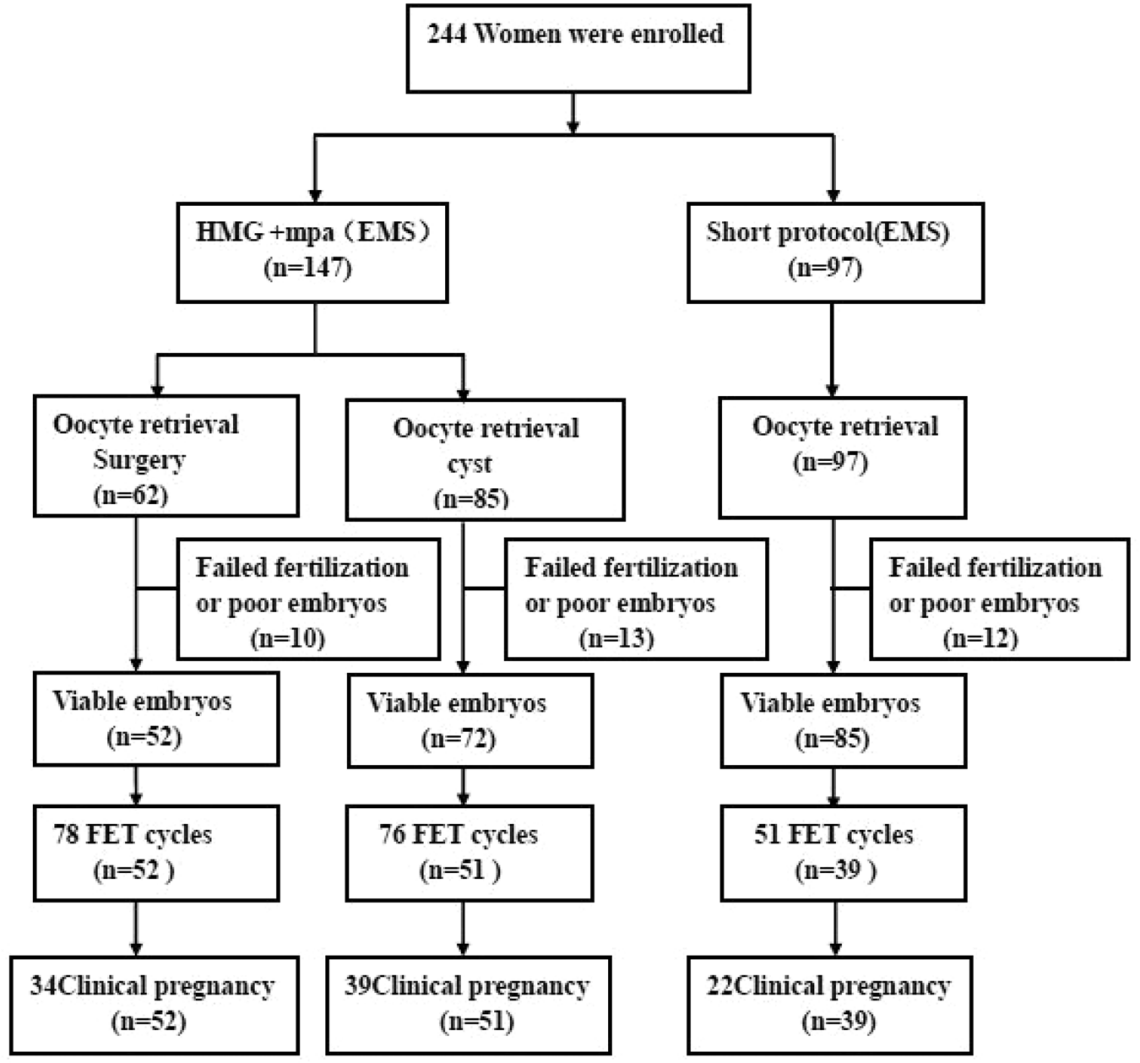
Patient flowchart.

Figure 1 shows a profile summary of the study. A total of 262 women were grouped into the surgery, aspiration, or short protocol groups according to a diagnosis of “endometriosis surgery,” “presence of endometrioma”, and the protocol they received. Among them, 244 women completed oocyte retrieval cycles (surgery group, 71 cycles in 62 patients; aspiration group, 90 cycles in 85 patients; short protocol group, 101 cycles in 97 patients) and 142 women completed FET cycles (surgery group, 78 cycles in 52 patients; aspiration group, 76 cycles in 51 patients; short protocol group, 51 cycles in 39 patients).

There was no significance among the three groups in terms of age, BMI, number of antral follicles, duration of infertility, and primary infertility rate (Table 1). Regression analyses revealed no effect of these factors on pregnancy outcome.

**Table 1:** Characteristics of women with endometriosis undergoing IVF/ICSI.

Characteristics	Surgery Group (EMS surgery 62)	Aspiration Group (EMS cyst 85)	Short protocol (EMS 97)	P1 Values	P2 Values	P3 Values	P Values
Age, years	31.4 ± 3.7	31.5 ± 4.0	31.6 ± 4.0	0.84	0.863	0.841	0.928
BMI (kg/m^2^)	21.0 ± 2.8	20.7 ± 2.5	20.8 ± 2.4	0.180	0.832	0.133	0.273
Duration of infertility, years	3.4 ± 2.4	3.0 ± 2.1	3.0 ± 1.8	0.227	0.894	0.193	0.336
Day 3 measures							
Basal FSH, IU/L	6.13 ± 1.25	5.83 ± 1.26	5.61 ± 1.82	0.014	0.261	0.182	0.049
Basal LH, IU/L	3.30 ± 1.34	3.54 ± 1.25	3.25 ± 1.63	0.832	0.205	0.360	0.425
Basal E_2_, pg/mL	35.22 ± 18.03	40.25 ± 15.50	35.04 ± 15.51	0.946	0.036	0.074	0.077
Basal P, ng/mL	0.34 ± 0.13	0.27 ± 0.10	0.26 ± 0.13	0.449	0.124	0.532	0.302
Primary infertility,%	72.6 (45/62)	67.1 (57/85)	73.3 (74/101)	0.421	0.954	0.473	0.616
Antral follicle count, n	8.8 ± 3.5	9.4 ± 3.8	9.3 ± 3.3	0.476	0.817	0.365	0.645

^#^P1: Surgery group vs. short protocol group.

^*^P2: Aspiration group vs. short protocol group.

^$^P3: Surgery group vs. Aspiration group.

BMI = body mass index, EMS = endometriosis, FSH = follicle stimulating hormone, LH = luteinizing hormone, E_2_ = estrogen, P = progesterone.

### Hormone profiles during treatment

**Figure 2:**
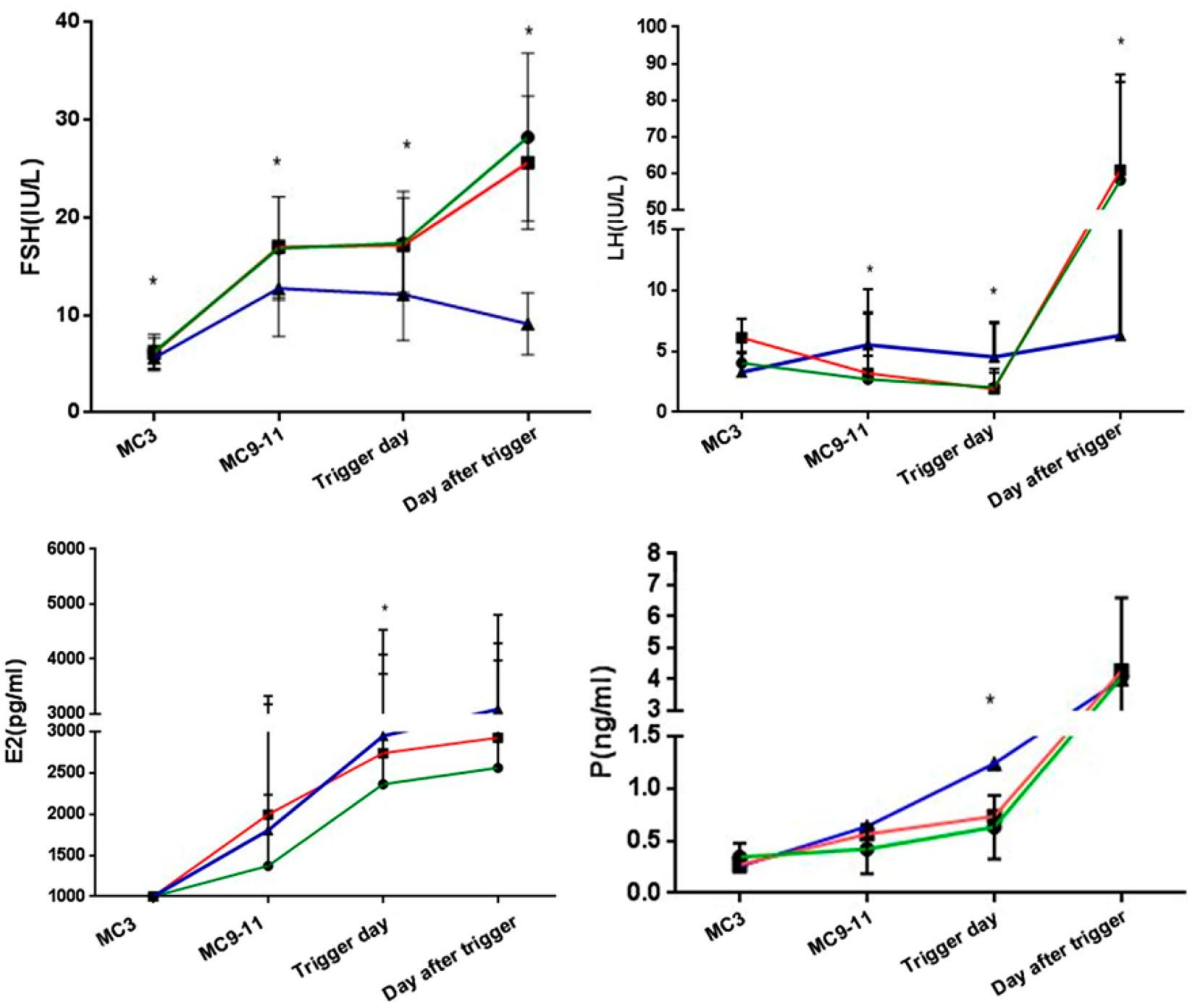
Hormone profiles of the medroxyprogesterone acetate (MPA) + human menopausal gonadotrophin (hMG) protocol in the three groups with trigger by gonadotropin-releasing hormone agonist (GnRH-a) or human chorionic gonadotrophin (hCG). There were temporal associations among circulating levels of follicle stimulating hormone (FSH), luteinizing hormone (LH), estrogen (E_2_), and progesterone (P). The green line shows the surgery group, the red line refers to the aspiration group, and the blue line refers to the short protocol group. The results are presented as mean ± SD. *P < 0.05 at the same time point.

The values of circulating levels of FSH, LH, E_2_, and P in the study groups are presented in Fig. 2. FSH levels increased significantly after hMG administration and kept steady during ovarian stimulation. After trigger, the average FSH levels increased (surgery group: 28.26 ± 8.60 IU/L; aspiration group: 25.62 ± 6.82 IU/L; short protocol group: 9.11 ± 3.18 IU/L; P < 0.001). The levels of FSH on the day after trigger were higher in the surgery and aspirations group than in the short protocol group owing to double trigger or HCG trigger alone. The LH values gradually decreased during ovarian stimulation, and the mean LH levels on the trigger day was significantly lower than the basal LH values in the two endometriosis groups (surgery group: 3.30 vs. 1.57 IU/L; aspiration group: 3.54 vs. 1.67 IU/L; short protocol group: 5.61 vs. 4.67 IU/L) and then increased significantly (surgery group: 58.15 IU/L; aspiration group: 60.90 IU/L; short protocol group: 9.11 IU/L; P < 0.001) 10 h after trigger. Serum E_2_ values showed a gradual increase accompanying the growth of follicles during ovarian stimulation after trigger (surgery group: 2561.20 pg/mL; aspiration group: 2928.21 pg/mL; short protocol group: 3086.22 pg/ml, P = 0.306). P levels remained low during ovarian stimulation; during MC9-11, the levels of P on the trigger day in the two endometriosis groups were lower than in the short protocol group (surgery group: 0.54 ng/mL; aspiration group: 0.58 ng/mL; short protocol group: 1.24 ng/ml; P = 0.001) and increased significantly after trigger (surgery group: 4.08 ng/mL; aspiration group: 4.27 ng/mL; short protocol group: 4.00 ng/ml; P = 0.711) (Fig. 2).

The LH levels remained low during ovarian stimulation, and there was an absence of concealed or premature LH peak. No patients had premature ovulation.

### Ovarian stimulation, follicle development, and oocyte performance

The short protocol group was characterized by a lower stimulation dose of hMG (surgery group: 1886.14 IU; aspiration group: 1888.19 IU; short protocol group: 1562.38 IU; P < 0.001), but the stimulation durations showed significant differences among the three groups (surgery group: 9.04 days; aspiration group: 8.89 days; short protocol group: 10.92 days). The number of follicles with diameter >10 mm (surgery group: 9.21; aspiration group: 9.71; short protocol group: 10.88) or 14 mm (surgery group: 6.74; aspiration group: 7.04; short protocol group: 9.98) were similar in the three groups. No significant differences were found in the oocyte maturation rate, high quality embryo (D3 8-cell grade I and II), or viable embryo rate per oocyte retrieved among the 3 groups (P > 0.05). The maturation rate was significantly higher in the two endometriosis groups than in the short protocol group (87.53% vs. 85.83% vs. 81.86%, P = 0.001). The interval from trigger to oocyte retrieval were significantly longer in the study groups than in the short protocol group (36.00 h vs. 36.03 h vs. 35.30 h, P = 0.032), but the oocyte retrieval rate showed no differences in the three groups. The fertilization and cleavage rates were similar in the three groups. The cycle cancellation rate due to no viable embryos was not different in the three groups (surgery group: 14.29%; aspiration group: 14.29%; short protocol group: 11.88%; P > 0.05). No patients experienced moderate or severe OHSS in the study group (Table 2).

**Table 2:** Cycle characteristics of the women in the three groups undergoing IVF/ICSI.

Characteristics	Surgery Group (EMS surgery 62)	Aspiration Group (EMS cyst 85)	Short protocol (EMS 97)	P1 Values	P2 Values	P3 Values	P Values
hMG dose (IU)	1886.1 ± 438.5^#^	1888.2 ± 466.3^*^	1562.4 ± 531.1^*#^	<0.001	<0.001	0.979	<0.001
hMG duration, days	9.0 ± 1.6^#^	8.9 ± 1.7^*^	10.9 ± 2.1^*#^	0.001	0.003	0.607	0.001
hMG dose per follicle, IU	259.4 ± 91.3^#^	280.6 ± 98.9^*^	200.6 ± 179.6^*#^	0.006	<0.001	0.326	<0.001
No. of >10-mm follicles on the trigger day	9.2 ± 5.4	9.7 ± 5.4	10.9 ± 6.0	0.393	0.750	0.585	0.693
No. of >14-mm follicles on the trigger day	6.7 ± 4.6	7.0 ± 4.7	10.0 ± 6.3	0.313	0.515	0.693	0.585
No. of oocytes retrieved, n	7.5 ± 5.2	8.1 ± 4.7	9.5 ± 5.0	0.075	0.243	0.493	0.187
No. of D3 top-quality embryos, n	2.7 ± 2.4	2.9 ± 2.5	2.8 ± 2.5	0.788	0.798	0.620	0.884
No. of viable embryos, n	2.9 ± 2.0	3.0 ± 2.4	3.4 ± 2.5	0.157	0.281	0.685	0.323
Interval from trigger to oocyte retrieval, hours	36 ± 0.64^#^	36.03 ± 0.58^*^	35.30 ± 1.63^#*^	0.041	0.01	0.569	0.032
Oocyte retrieval rate, %	67.33% (1251/1858)	69.25% (734/1060)	66.86% (904/1352)	0.781	0.214	0.286	0.427
Mature oocyte rate, %	87.53%(1095/1251)^#^	85.83% (630/734)^*^	81.86%(740/904)^#*^	<0.001	0.031	0.279	0.001
Fertilization rate, %	72.66% (909/1251)	69.75% (512/734)	71.46% (646/904)	0.539	0.451	0.166	0.885
Cleavage rate, %	97.48% (886/909)	97.27% (498/512)	95.80% (627/646)	0.622	0.834	0.817	0.085
Viable embryo rate per oocyte retrieved, %	38.05% (476/1251)	37.47% (275/734)	37.83% (342/904)	0.918	0.879	0.796	0.967
High quality embryo rate per oocyte retrieved, %	36.53% (457/1251)^#^	36.24% (266/734)^*^	31.64% (286/904)^#*^	0.018	0.05	0.897	0.043
Cancellation rate, %	14.29% (10/70)	14.29% (13/91)	11.88% (12/101)	0.650	0.671	0.990	0.856

EMS = endometriosis, MPA = medroxyprogesterone acetate, HMG = human menopausal gonadotropin.

^#^P1: surgery group vs. short protocol group.

^*^P2: aspiration group vs. short protocol group.

^$^P3: surgery group vs. aspiration group.

### Pregnancy outcomes in FET cycles

FET pregnancy outcomes in the three groups are presented in Table 3. A total of 142 women (surgery group: 52; aspiration group: 51; short protocol group: 39) in the three groups completed a total of 205 FET cycles (surgery group: 78; aspiration group: 76; short protocol group: 101). The implantation rate (surgery group: 31.43%; aspiration group: 35.56%; short protocol group: 26.80%), clinical pregnancy rate (surgery group: 43.59%; aspiration group: 51.32%; short protocol group: 43.14%), miscarriage rate, multiple pregnancy rate, and ongoing pregnancy rate were similar among the groups (all P > 0.05), which indicated that the embryos in the different groups shared similar development potential (Table 3).

**Table 3:** Pregnancy outcomes from frozen-thawed embryo transfers in the three groups.

Characteristics	Surgery Group (EMS surgery 62)	Aspiration Group (EMS cyst 85)	Short protocol (EMS 97)	P Values
No. of patients	52	51	39	
No. of FET cycles	78	76	51	
Pregnancy outcome of FET				
Biochemical pregnancy rate per transfer	50% (39/78)	57.89% (44/76)	49% (25/51)	0.515
Clinical pregnancy rate per transfer	43.59% (34/78)	51.32% (39/76)	43.14% (22/51)	0.548
Implantation rate	31.43% (44/140)	35.56% (48/135)	26.80% (26/97)	0.392
Miscarriage rate	2.94% (1/34)	15.38% (6/39)	9.09% (2/22)	0.139
Ectopic pregnancy rate	0% (0/26)	7.69% (3/39)	4.55% (1/22)	0.364
Multiple pregnancy rate	23.08% (6/26)	23.08% (9/39)	22.73% (5/22)	0.990
Ongoing pregnant rate per transfer	43.42% (33/76)	39.47% (30/76)	37.25% (19/51)	0.786

Abbreviations: EMS = endometriosis, FET = frozen-thawed embryo transfer.

### Logistic regression of clinical pregnancy outcome

Logistic regression analysis with clinical pregnancy as the dependent variable and independent variables of the baseline characteristics of the patients and the group allocation revealed no significant effect of basic characteristics on the outcome (Table 4).

**Table 4:** Logistic regression of pregnancy outcome.

Baseline Parameter	OR value	P value	95% CI
Age (y)	1.035	0.199	0.982–1.091
BMI (kg/m2)	1.035	0.419	0.952–1.126
Duration of infertility (y)	0.957	0.578	0.819–1.118
Number of previous attempts	0.89	0.173	0.753–1.052
FSH (IU/L)	0.968	0.823	0.731–1.283
LH (IU/L)	1.075	0.413	0.904–1.279
E_2_ (pg/mL)	0.985	0.888	0.968–1.002
P (ng/mL)	0.892	0.851	0.290–2.949
Endometrial thickness	0.964	0.658	0.821–1.132
Three different groups	0.919	0.795	0.487–1.736

BMI = body mass index, CI = confidence interval, OR = odds ratio FSH = follicle stimulating hormone, LH = luteinizing hormone E_2_ = estrogen, P = progesterone.

## Discussion

The aim of this study was to evaluate the use of MPA cotreatment with hMG for women with ovarian endometriosis undergoing COH and to investigate the outcome after subsequent FET. To our knowledge, no previous studies have reported using the combination of hMG and MPA as a controlled ovarian stimulation regimen in IVF cycles for women with endometriosis who have normal ovarian function. The results strongly suggest that mature oocyte rate and D3 high quality embryo rate were better in the two endometriosis groups using MPA compared with the short protocol group. The rates of oocyte retrieval, fertilization, cleaved embryos retrieved, cancellation and the pregnancy results after FET in the study group were similar to those in the short protocol. These results suggest that the quality of oocytes and embryos derived from patients with severe endometriosis receiving the MPA regimen were not affected by endometriosis, regardless of whether the patients underwent surgery or had endometriotic cysts. This could be because: (1) the ovarian endometriosis itself is unlikely to affect the quality of oocytes; (2) the sample size was small; and (3) the use of progesterone may improve the egg or embryo quality of women with endometriosis. This strongly suggests that chocolate cyst surgery or the cyst itself is unlikely to affect the quality of oocytes and embryos. This may be because progesterone can improve the peritoneal and ovarian microenvironment in patients with endometriosis, so as to improve the inflammatory reaction, thereby improving quality of oocytes and embryos. At the same time, in terms of pregnancy outcome, there were no significant differences in implantation rate and clinical pregnancy among the three groups. These results are supported by previous studies that found no difference among endometrial preparation protocols^38,39^. This again strongly suggests that for endometriosis patients with normal ovarian function, a progesterin-primed ovarian stimulation regimen is an appropriate choice.

Women with advanced endometriosis have a reduced pregnancy rate compared with women with tubal factor infertility due to reduced fertilization and implantation rates^14–18^. Furthermore, patients with stage III or IV endometriosis have poorer outcomes after assisted reproductive procedures than women with stage I or II endometriosis^14–18^. On the other hand, some investigators found no differences in pregnancy rates when comparing patients with and without endometriosis^19–25^. Adverse effects of endometriosis on oocyte and embryo quality, as well as on implantation rates, have been reported by several investigators^14–18,40^, but this negative impact was not found in the present study. Many factors may contribute to those results. Firstly, in many previous studies, embryos were transferred during fresh cycles, but in the present study, since patients with endometriosis often have low-quality oocytes, particular care have been taken when selecting the embryos. Consequently, only high-quality embryos were used in FET cycles, which excluded the effect of embryo quality on the implantation rate and pregnancy outcomes in women with severe endometriosis. Secondly, the effects of down-regulated GnRH-a and high doses of E_2_ and progesterone on endometrial receptivity were further excluded. Thirdly, an endometrial cyst suggests active endometriosis, but the implantation rate was not affected, which means that the endometrial receptivity and the potential of high-quality embryos were not affected in FET cycles. Taken together, these results suggest that MPA is an effective oral alternative for women with advanced endometriosis undergoing COH, which will help to establish a convenient user regimen in combination with FET.

Nevertheless, two main differences with previous studies^14–25,40^ should be underlined. First, the patients included here were all patients with ovarian endometriosis and some patients in the surgery group also had peritoneal endometriosis. Secondly, all patients had normal ovarian function. Indeed, patients with poor ovarian function were excluded in order to avoid the influence of the ovaries on embryo and pregnancy outcomes.

The exact mechanisms of progestin action and the effect of progestin administration on the hypothalamic-pituitary-ovarian (HPO) axis for premature LH surge suppression and premature ovulation remain to be explored. Progestin can block estradiol-induced LH surges by preventing the activation of the GnRH surge induction system by estradiol. The mechanisms of action of progestin on pituitary LH and FSH discharges are mediated by the interaction of estradiol, progestin, and GnRH neurosecretory system^41,42^. There was a trend toward progressively deeper serum LH suppression during the hMG and MPA protocol. This may have contributed to the higher amount of hMG used, and this protocol used in this study worked well, with regard to prevention of a premature LH surge. Nevertheless, the duration of hMG administration and the hMG dose were higher in the study group than in the short protocol group, which is consistent with previous studies^43,44^. The extent of pituitary suppression during COH could be responsible for this observation. Indeed, LH levels on the trigger day in the MPA groups were significantly lower than in the short protocol group. In addition, the high progesterone environment could have a direct influence on the function of ovarian granulosa cells.

MPA showed no detrimental effect on oocytes and embryos development potential in normally ovulating women in our previous reports^32,33^, which has been recognized by others^45^. In the present study, MPA cotreatment with hMG did not reduce oocyte quality or the fertilization rate from endometriosis patients compared to the short protocol. MPA administration showed good ovum and embryo quality. Progestogens may prevent implantation and growth of regurgitated endometrium inhibiting the expression of matrix metalloproteinases and angiogenesis, and they have several anti-inflammatory *in vitro* and *in vivo* effects that may reduce the inflammatory state generated by the metabolic activity of the ectopic endometrium, and the consequent immune response. Endometriosis is a disease in which the entire immune system is profoundly altered. This means that the anti-angiogenetic, anti-inflammatory, and immunomodulatory effects, down-regulation of endometrial cell proliferation, and increased apoptotic activity may all constitute a convincing rational basis for the use of progestogens in endometriosis because they influence granulosa cells and improve the quality of oocytes and embryos, which in return, improve pregnancy outcomes^46,47^. MPA may have anti-inflammatory properties in women with endometriosis due to the reduction of chemokine synthesis. The administration of MPA for 8 days increases the expression of progesterone receptor (PR)^48^. Progesterone is also effective in inducing apoptosis in endometrial and endometriotic cells through the inhibition of Bcl-2 and nuclear factor-κB^48^. Progestogens alone are generally well-tolerated, have a more limited metabolic impact than danazol or GnRH agonists, are inexpensive, and may be used on a long-term basis^42,49–51^. In addition, endometriosis and adenomyosis are frequently encountered together^52–54^, but patients with adenomyosis were excluded from the present study, probably contributing to the good outcomes observed here.

In our IVF procedure, the overall cancellation rate was 14.29% in the MPA groups and 13.10% in the short protocol group. The procedure was cancelled when no good-quality embryos could be obtained and frozen. The results suggest that endometriosis does not affect embryo quality and the related parameters of pregnancy, as indicated by the fertilization rate, embryo quality, implantation rate, pregnancy rate, independent of active ovarian endometrioma or surgery treatment.

Nevertheless, a major limitation of our study is that it was a retrospective analysis; a well-designed, prospective, randomized study in severe advanced endometriosis patients undergoing FET and embryos derived from different parallel controls without MPA should be performed. Another probable bias is that we were unable to assess the nature and severity of peritoneal endometriosis in the aspiration group, but merely according to the pathologic results aspirated fluid of the cyst, diameter, and number of endometriomas. Therefore, the precise stage of endometriosis in the aspiration group remained unknown. Those patients were staged III-IV according to the revised American Fertility Society (AFS) classification of endometriosis^35^, although this stage were not evaluated by laparoscopy or laparotomy. Furthermore, patients with stage I-II were not included. Finally, the change of granulosa cells, biochemical levels, and various immune factors during the MPA protocol should be detected in future studies.

In conclusion, the administration of MPA in COH showed no cases of premature LH surge, and a similar pregnancy outcome was achieved in patients with ovarian advanced endometriosis undergoing IVF/ICSI compared to the short protocol. The new strategy provides a novel insight into an alternative to the conventional regimen. Progestogens alone or combined with estrogens are generally well-tolerated, may have a more limited metabolic impact than danazol or GnRH agonists, and may be used repeatedly^49–51^. The role of MPA in COH appears to be promising although many questions remain to be answered including the extent of progestin priming, the possible influence on embryo development potential and the stable hormonal milieu. Given their good tolerability, minor metabolic effects, and low cost, progestogens must therefore be considered the COH of choice.
